# The Thermal Stress Problem of Bimodular Curved Beams under the Action of End-Side Concentrated Shear Force

**DOI:** 10.3390/ma16155221

**Published:** 2023-07-25

**Authors:** Xiao-Ting He, Xin Wang, Meng-Qiao Zhang, Jun-Yi Sun

**Affiliations:** 1 School of Civil Engineering, Chongqing University, Chongqing 400045, China; 202216131258t@stu.cqu.edu.cn (X.W.); 20165039@cqu.edu.cn (M.-Q.Z.); sunjunyi@cqu.edu.cn (J.-Y.S.); 2 Key Laboratory of New Technology for Construction of Cities in Mountain Area (Chongqing University), Ministry of Education, Chongqing 400045, China

**Keywords:** thermal stress, bimodular materials, curved beams, tension and compression, concentrated shear force

## Abstract

A bimodular material is a kind of material that presents two elastic moduli in tension and compression. In classical thermoelasticity, however, the bimodular material is rarely considered due to its complexity in analysis. In fact, almost all materials will present, more or less, bimodular characteristics, and in some cases, the mechanical properties of materials cannot be fully utilized simply by ignoring the bimodular characteristics. In this study, the thermal stress problem of bimodular curved beams under the action of end-side concentrated shear force is analytically and numerically investigated, in which the temperature rise modes in a thermal environment are considered arbitrary. Using the stress function method based on compatibility conditions, a two-dimensional solution of thermoelasticity of the bimodular curved beam subjected to end-side concentrated shear force was obtained. The results show that the solution for a bimodular curved beam with a thermal effect can be reduced to that of a bimodular curved beam without a thermal effect. At the same time, the numerical simulation for the problem verifies the correctness of the theoretical solution. The results may serve as a theoretical reference for the refined analysis and optimization of curved beams in a thermal environment.

## 1. Introduction

Curved beams, as one common load-bearing and connecting component, are widely used in the fields of mechanical engineering and civil engineering. With the development of material technology, the consideration of the material characteristics of these components is no longer satisfied by the traditional assumption of a single modulus of elasticity [1,2,3]. In the academic and engineering fields, the bimodular characteristics of materials have gradually been paid attention. On the other hand, these curved beam components sometimes need to serve in certain high-temperature environments, so it is necessary to investigate their problems with thermal stress. In this study, we used theoretical and numerical methods to investigate the thermal stress problems of bimodular curved beams in order to make a valuable contribution to the thermal stress field of bimodular materials and structures. To this end, the next review will be conducted from the following aspects: we will begin with the bimodular materials model and the analysis of bimodular structures, next will be the development of the theory of thermoelasticity, and lastly, on the basis of the review, the shortcomings of the existing research are analyzed to propose the problem to be solved in this study.

Many studies have indicated that some materials [4,5,6], such as graphite, ceramics, rubber, concrete, and certain biomedical materials, under the same tensile and compressive stresses have different tensile and compressive strains. Jones [1] referred to these materials as bimodular or multimodulus materials. In the theoretical analysis of the engineering field, two kinds of bimodular material models are widely used. One is the Bert model [2], based on a positive-negative signature of longitudinal fiber strain. This model is generally used when analyzing orthotropic materials and laminated composites [7,8,9]. Another is the Ambartsumyan model, which was established on the principle of positive and negative signs of principal stress [3], and is mainly suitable for the analysis of isotropic materials. Existing studies have shown that the application of the Ambartsumyan model to structural analysis is of special interest because it is the judgment of the principal stress that determines whether a certain point in the structure is tensile or compressive. Our work is thus based on the latter model of principal stress.

**Figure 1:**
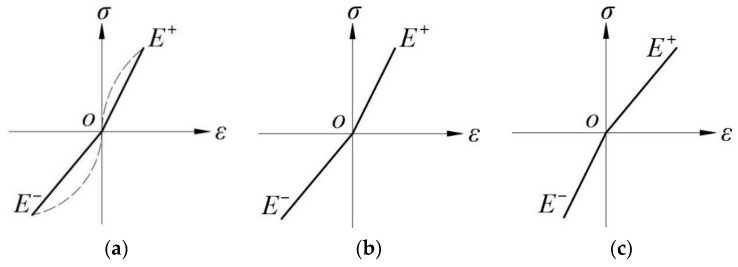
Ambartsumyan bimodular model. (**a**) the real case; (**b**) *E*^+^ > *E*^−^; and (**c**) *E*^+^ < *E*^−^.

For the description of the stress-strain relation, two broken straight lines were used by Ambartsumyan [3] to linearize a real bimodular model, which was originally nonlinear, as shown in Figure 1, where (a) is the real case and (b) and (c) are the bilinear models. In Figure 1, the principal stress is *σ*, and the principal strain is *ε*. For this model, the basic assumptions are as follows. (1) The research object is continuous, elastic, isotropic, and homogeneous. (2) The material satisfies the small deformation assumption. (3) When the material is stretched along a certain principal direction, the Young’s modulus is *E*^+^, and the Poisson’s ratio is *μ*^+^. When the material is compressed, the corresponding quantities are *E*^−^ and *μ*^−^. (4) For three-dimensional problems, when the three principal stresses are all positive or negative, the equilibrium equation, geometric equation, and physical equation are basically the same as those of classical elasticity. However, when the signs of the three principal stresses are different, except for the physical equation, the remaining two equations are the same as the classical elastic equation. (5) *μ*^+^/*E*^+^ = *μ*^−^/*E*^−^, this ensures the symmetry of the compliance matrix in the application of the finite element method.

According to the above bimodular material model, the constitutive relationship of the bimodular material is based on the positive and negative signs of the determined principal stresses, thus meaning that the principal stresses are known in advance. In the vast majority of cases, however, the principal stress is usually obtained as a final result rather than as a known condition before the solution. In addition, it is difficult to describe the elastic coefficients experimentally under complex stress states. Analytical solutions can be obtained in some simple cases, although they only deal with bending beams and plates [10,11,12]. In complex problems, we have to resort to finite element methods using iterative techniques [13,14,15,16].

In the theory of thermoelasticity [17], it is generally assumed that the material constituting an elastic body is completely elastic, homogeneous, and isotropic, so the conventional analysis in classical elasticity [18] can also be used for thermoelastic analysis. For example, the Lame equation in thermoelasticity is formulated with the displacement component as the basic variable and solved by the conventional method of classical elasticity. However, if the bimodular effect of the material is newly incorporated into the original thermoelasticity, the existing analysis will more or less encounter difficulties. Thus, for a specific problem, our work may be focused on what and how much the impact of the bimodal effect on existing results is.

With the development of thermoelastic theory, some generalized thermoelastic models have been proposed for transient responses in many applications, such as low temperature and ultra-fast laser heating, where the classical thermoelastic theory fails. Some representative theories in this regard are shown in [19,20,21,22]. It is important to note that Green and Lindsay’s theory has been used in many types of media, among which Marin et al. [23] used it in dipolar thermoelastic bodies. On the other hand, in addition to the development of the theory itself, it is also very important to apply the theory to analyze engineering components, or more specifically, to analyze the thermoelastic behavior of engineering structures, such as nanobeams [24], microbeams [25], composite beams [26], and laminated beams [27]. Obviously, bimodular material beams, including straight and curved beams, should also be investigated in thermoelastic analysis of structures.

Following this demand, some scholars have carried out research on bimodular beams in a thermal environment [28,29,30,31]. Wen et al. [28] first obtained a two-dimensional thermoelastic solution of a bimodular beam under thermal and mechanical loads, where the Duhamel similarity theorem was used to transform the thermoelastic problem into a pure elastic problem. For the thermal stress problem of the bimodular functionally graded beam, the displacement method based on the Duhamel similarity theorem is no longer applicable, so Xue et al. [29] used the stress method to obtain one-dimensional and two-dimensional thermal stress solutions under different temperature rise modes. For metal bars, Guo et al. [30] used the commonly used strain suppression method to derive a one-dimensional thermal stress expression and, at the same time, used the Duhamel similarity theorem to derive the two-dimensional thermoelastic solution. Unfortunately, the above works are limited to straight beams; relatively little research has been found on curved beams. As indicated above, curved beams are important components of a special shape with an initial curvature. Compared to the analysis of straight beams, the analysis of curved beams is more or less complicated due to the presence of the initial curvature.

**Figure 2:**
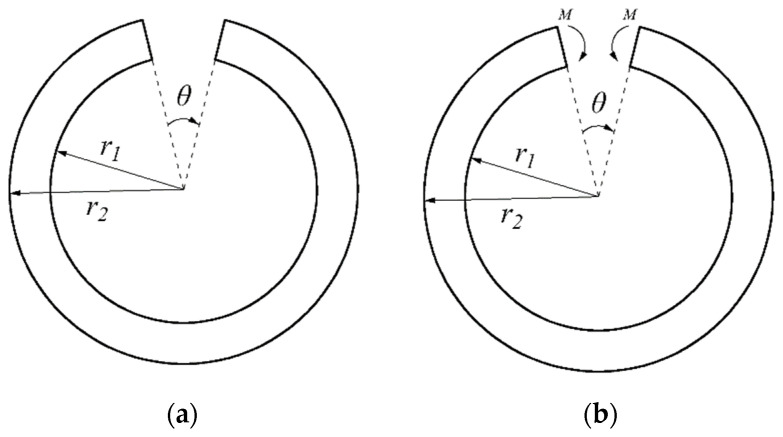
Welding of a bimodular material ring with initial stresses. (**a**) a ring with a gap; (**b**) the mechanical model.

**Figure 3:**
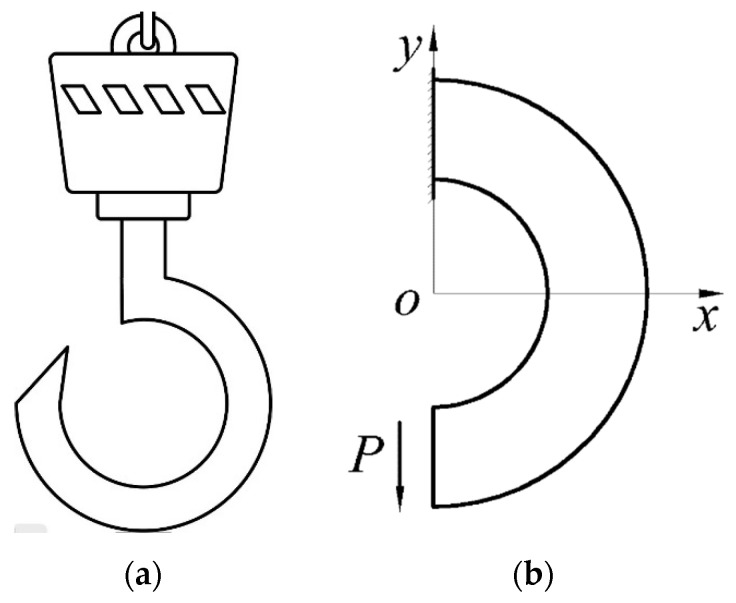
Hook made by bimodular materials in a thermal environment. (**a**) sketch of a hook; (**b**) mechanical model.

More recently, He et al. [31] first investigated a bimodular curved bar under pure bending in a thermal environment; the application of this problem may be easily found in mechanical engineering. For example, if a certain portion between two adjacent cross-sections of a ring is cut out (see Figure 2a), joining the ends of the ring again by welding or another means gives a ring with an initial stress, that is, there is stress in the ring in the absence of an external force. In Figure 2, *r*_1_ and *r*_2_ are the inner radius and outer radius of the ring, respectively, and *θ* denotes the small angle measuring the portion of the ring that was cut out. Obviously, the closing of the ring requires the application of two bending moments at the two ends of the ring, as shown in Figure 2b. The real problem finally returns to a bimodular curved bar under pure bending in a thermal environment, and the stress state is axisymmetric. But more general cases may be found in lifting machines and other cases, for example, a hook made from bimodular materials in a thermal environment, as shown in Figure 3a. In this case, the mechanical model is simplified as a curved beam with an end-side concentrated shear force, as shown in Figure 3b. Obviously, due to the existence of concentrated shear force, the solving problem has not been an axisymmetric one; more importantly, the corresponding solving method has thus changed much.

In our previous study aiming at the pure bending problem [31], it was found that the thermoelastic plane stress problem with a bimodular effect can also be transformed into a purely elastic problem under known body forces and known surface forces, which is the familiar Duhamel similarity theorem. However, once the beam is no longer under pure bending but under transverse bending, the axisymmetric stress state in pure bending is no longer maintained, as shown in Figure 3. In this case, the previous method based on displacement is no longer applicable, and a new solving method must be resorted to.

Aiming at this more general case of curved beams, in this study, we analytically and numerically investigated a bimodular curved beam under the action of end-side concentrated shear force in a thermal environment. To this end, the whole paper is organized as follows. The problem is briefly described in Section 2 first, and the mechanical model established on the subarea of tension and compression is also given. Section 3 is the analytical solving process of the mechanical problem proposed, using the stress function method based on compatibility conditions but not the displacement method. In Section 4, the numerical simulation is conducted to verify the correctness of the theoretical solution. The bimodular effect on stress distribution is discussed in Section 5, and Section 6 contains the concluding remarks.

## 2. Problem

**Figure 4:**
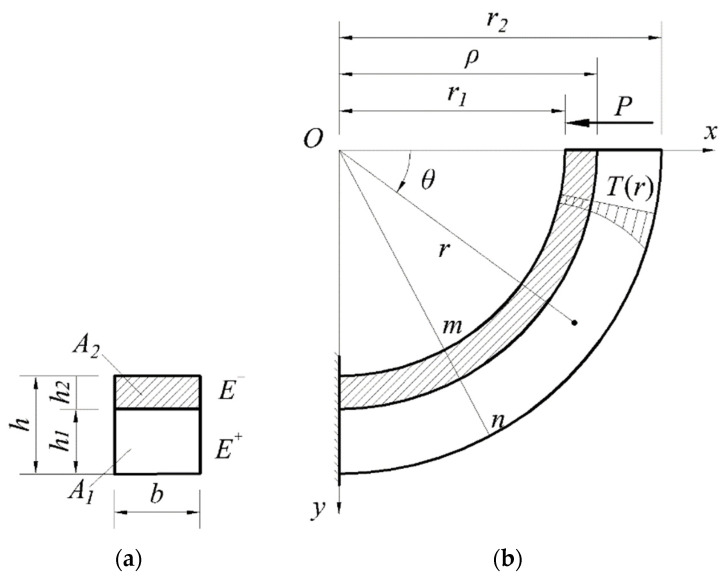
A bimodular curved beam under concentrated shear force in a thermal environment. (**a**) the cross-section; (**b**) the whole curved beam.

A curved beam with a rectangular section type was subjected to the concentrated shear force *P* at one end and was fully fixed at another end, as shown in Figure 4. The polar coordinate system *rOθ* and the rectangular coordinate system *xOy* were combined to describe this problem. For any point of the beam, its polar radius was denoted by *r* and its polar angle by *θ,* whose positive rotation direction was defined as: from the positive half *x*-axis to the positive half *y*-axis; that is, the positive rotation direction was clockwise, as shown in Figure 4b. The inner radius of the curved beam was denoted by *r*_1_, the outer radius by *r*_2_, and the curvature radius of the neutral layer by *ρ,* which was unknown at present. Due to the constraint of the structure, under a certain temperature field, the expansion and contraction caused by temperature changes will not be able to develop freely, resulting in the so-called thermal stress. Without loss of generality, we assume the temperature change is only along the radial direction, this is, *T* = *T*(*r*); in fact, this simple case is also very common in real problems. For example, in a ring or cylinder in a thermal environment in mechanical engineering, its heat distribution generally changes along the radial direction; thus, there are many thermal phenomena like this. In Figure 4b, *mn* stands for any cross-section whose section type is as shown in Figure 4a, in which the height of the cross-section is denoted by *h* and the width of the cross-section by *b*. Bounded by the neutral layer, the whole beam, or the cross-section, was divided into two parts; the bottom of the cross-section, or the outer part of the whole beam, was tensile, while the top of the cross-section, or the inner part of the whole beam, as shown by the shadowed area, was compressive, as shown in Figure 4. In Figure 4a, *h*_1_ and *h*_2_ denote the tensile height and the compressive height of the section, respectively; *A*_1_ and *A*_2_ denote the tensile area and the compressive area of the cross-section, respectively; *E*^+^ and *E*^−^ denote the tensile modulus and compressive one, respectively.

The basic assumptions used in this study are as follows. (i) The initial neutral layer depends only upon the bending moment produced by shear force, having nothing to do with the external thermal environment. (ii) Like the common analysis for shallow beams, the bending is limited to in-plane small deflection bending without torsion. (iii) The temperature varies only along the radial direction of the curved beam, resulting in *T* = *T*(*r*), as indicated before. (iv) It is assumed that the material properties are independent of temperature, or alternatively, if they are dependent on temperature, we may take a constant average value to describe this dependency. The occurrence of creep, relaxation, and phase transformation of the material is not considered in this work. Among the four assumptions, only assumption (ii) aligns with the Euler-Bernoulli beam theory, while assumptions (i), (iii), and (iv) differ from the Euler-Bernoulli beam theory.

Under the action of end-side concentrated shear force, a further deflection inward will occur in the curved beam, thus forming compression for the inside part and tension for the outside part of the beam. The elastic modulus for the inside part was taken as *E*^−^ while the modulus for the outside part was taken as *E*^+^ accordingly, as indicated before. In this study, we neglected the differences between the tensile and compressive Poisson’s ratios. There were two main reasons for this practice. The first was that, if neglecting the difference in tension and compression, the whole derivation process was slightly simple, but another important reason was based on the fact that the influence of the Poisson’s ratio on the results is small; thus, the moderate simplification is rational, and many other works were based on this simplification. The same practice was followed for the line expansion coefficient α, also neglecting the differences in tension and compression. In a word, for modulus of elasticity, we considered *E*^+^ and *E*^−^; while for Poisson’s ratio and the line expansion coefficient, we only had *μ* and α.

For the realization of the tension-compression subarea, we needed to determine the position of the unknown neutral layer of the beam under pure bending first. The solution was obtained in our previous study [31], which gave:

(1) $$\frac{E^{+}}{E^{-}}(r_{2}-\rho )+\rho -r_{1}-\frac{E^{+}}{E^{-}}\rho \ln\frac{r_{2}}{\rho }-\rho \ln\frac{\rho }{r_{1}}=0.$$

If the values of *E*^+^, *E*^−^, *r*_1_, and *r*_2_ are given, we may use Equation (1) to determine the unknown neutral layer, that is, the curvature radius of this layer, *ρ*, and finally, the simplified mechanical model based on the subarea of tension and compression is thus constructed.

## 3. Theoretical Solution

### 3.1. Application of the Stress Function Method

First, we gave the physical equation of two-dimensional thermoelasticity. As indicated before, the problem was a representative plane stress problem concerning the thermal effect. Under a polar coordinate system, the strain components of a plane stress problem are denoted by ε*_r_*, ε*_θ_*, and *γ_rθ_*, and the corresponding stress components are denoted by σ*_r_*, σ*_θ_*, and *τ_rθ_*; the physical equation in two-dimensional thermoelasticity will give [18]:

(2) $$\left\{\begin{array}{l} \varepsilon_{r}=\frac{1}{E^{+/-}}\left(\sigma_{r}-\mu \sigma_{\theta }\right)+\alpha T\\ \varepsilon_{\theta }=\frac{1}{E^{+/-}}\left(\sigma_{\theta }-\mu \sigma_{r}\right)+\alpha T\\ \gamma_{r\theta }=\frac{2(1+\mu )}{E^{+/-}}\tau_{r\theta } \end{array}\right.,$$

in which α is the thermal expansion coefficient, *T* is the temperature change, *μ* is the Poisson’s ratio, and *E*^+/−^ stands for the tensile Young’s modulus and compressive modulus. For the time being, there is no way to determine in advance whether the stress state of any point is tensile or compressive.

In this study, the stress function method was adopted to solve the thermoelastic problem. From Figure 4, it is easy to see that the bending moment acting on any cross-section of the beam is:

(3) M=Py=Prsinθ,

this means *M* is proportional to sin*θ*, while the normal stress σ_θ_ is proportional to *M*, and also due to $\sigma_{\theta }=\partial^{2}\varphi /\partial r^{2}$, in which *φ* is the stress function; thus, we may prescribe the stress function is also proportional to sin*θ*, this gives:

(4) $$\varphi^{+/-}(r,\theta )=f_{1}^{+/-}(r)\sin\theta +f_{2}^{+/-}(r),$$

in which, $f_{1}^{+/-}(r)$ and $f_{2}^{+/-}(r)$ are two undetermined functions with respect to *r*. The superscript ‘+/−’ denotes the so-called tension and compression, and in some cases, they will naturally separate; this is easily seen in our next application to boundary conditions as well as continuity conditions. In fact, there is an implicit assumption here that due to *T* = *T*(*r*), the corresponding stress function in Equation (4) is also axisymmetric, that is, $f_{2}^{+/-}(r)$ holds true. If a more complicated temperature distribution is considered here, for example, *T* = *T*(*r,θ*), the corresponding stress function in Equation (4) should be assumed as $f_{2}^{+/-}(r,\theta )$. In addition, the structural form of the stress function shows that the final stress is a superposition of two effects, that is, the load effect and the temperature effect. In the stress function, the first term, $f_{1}^{+/-}(r)\sin\theta$, stands for the stress from the external load effect, while the second term, $f_{2}^{+/-}(r)$, represents the temperature effect, and the two effects are independent of each other. Specifically, if we let $f_{2}^{+/-}(r)$ be zero, we may obtain the solution of a bimodular curved beam without a thermal effect. Or vice versa, if we let $f_{1}^{+/-}(r)\sin\theta$ be zero, obviously, we may obtain the solution of a bimodular curved beam without a load effect.

The stresses may be expressed in terms of the stress function as:

(5) $$\left\{\begin{array}{l} \sigma_{r}{}^{+/-}=\frac{1}{r}\frac{\partial \varphi^{+/-}}{\partial r}+\frac{1}{r^{2}}\frac{\partial^{2}\varphi^{+/-}}{\partial \theta^{2}}\\ \sigma_{\theta }{}^{+/-}=\frac{\partial^{2}\varphi^{+/-}}{\partial r^{2}}\\ \tau_{r\theta }{}^{+/-}=-\frac{1}{r}\frac{\partial^{2}\varphi^{+/-}}{\partial r\partial \theta }+\frac{1}{r^{2}}\frac{\partial \varphi^{+/-}}{\partial \theta }=-\frac{\partial }{\partial r}\left(\frac{1}{r}\frac{\partial \varphi^{+/-}}{\partial \theta }\right) \end{array}\right..$$

Substituting Equation (4) into Equation (5), we have

(6) $$\left\{\begin{array}{l} \sigma_{r}{}^{+/-}=\sin\theta \left[\frac{1}{r}\frac{df_{1}^{+/-}(r)}{dr}-\frac{1}{r^{2}}f_{1}^{+/-}(r)\right]+\frac{1}{r}\frac{df_{2}^{+/-}(r)}{dr}\\ \sigma_{\theta }{}^{+/-}=\sin\theta \frac{d^{2}f_{1}^{+/-}(r)}{dr^{2}}+\frac{d^{2}f_{2}^{+/-}(r)}{dr^{2}}\\ \tau_{r\theta }{}^{+/-}=\cos\theta \left[\frac{1}{r^{2}}f_{1}^{+/-}(r)-\frac{1}{r}\frac{df_{1}^{+/-}(r)}{dr}\right] \end{array}\right..$$

Also, substituting Equation (6) into Equation (2), the strains expressed in terms of stress function are

(7) $$\left\{\begin{array}{ll} \varepsilon_{r}^{+/-} & =\frac{1}{E^{+/-}}\left\{\sin\theta \left[\frac{1}{r}\frac{df_{1}^{+/-}(r)}{dr}-\frac{1}{r^{2}}f_{1}^{+/-}(r)\right]\right.\\ & +\left.\frac{1}{r}\frac{df_{2}^{+/-}(r)}{dr}-\mu \left[\sin\theta \frac{d^{2}f_{1}^{+/-}(r)}{dr^{2}}+\frac{d^{2}f_{2}^{+/-}(r)}{dr^{2}}\right]\right\}+\alpha T\\ \varepsilon_{\theta }^{+/-} & =\frac{1}{E^{+/-}}\left\{\sin\theta \frac{d^{2}f_{1}^{+/-}(r)}{dr^{2}}+\frac{d^{2}f_{2}^{+/-}(r)}{dr^{2}}\right.\\ & \left.-\mu \left[\frac{\sin\theta }{r}\frac{df_{1}^{+/-}(r)}{dr}-\frac{\sin\theta }{r^{2}}f_{1}^{+/-}(r)+\frac{1}{r}\frac{df_{2}^{+/-}(r)}{dr}\right]\right\}+\alpha T\\ \gamma_{r\theta }^{+/-} & =\frac{2(1+\mu )}{E^{+/-}}\cos\theta \left[\frac{1}{r^{2}}f_{1}^{+/-}(r)-\frac{1}{r}\frac{df_{1}^{+/-}(r)}{dr}\right] \end{array}\right..$$

In addition, the strains need to satisfy the following compatibility equation:

(8) $$\left(\frac{1}{r^{2}}\frac{\partial^{2}}{\partial \theta^{2}}-\frac{1}{r}\frac{\partial }{\partial r}\right)\varepsilon_{r}^{+/-}+\left(\frac{\partial^{2}}{\partial r^{2}}+\frac{2}{r}\frac{\partial }{\partial r}\right)\varepsilon_{\theta }^{+/-}-\left(\frac{1}{r^{2}}\frac{\partial }{\partial \theta }+\frac{1}{r}\frac{\partial^{2}}{\partial r\partial \theta }\right)\gamma_{r\theta }^{+/-}=0.$$

Substituting Equation (7) into Equation (8), we have

(9) $$\begin{array}{l} \frac{1}{E^{+/-}}\left(\frac{1}{r^{2}}\frac{\partial^{2}}{\partial \theta^{2}}-\frac{1}{r}\frac{\partial }{\partial r}\right)\left[\frac{\sin\theta }{r}\frac{df_{1}^{+/-}(r)}{dr}-\frac{\sin\theta }{r^{2}}f_{1}^{+/-}(r)\right]-\frac{\mu }{E^{+/-}}\left(\frac{1}{r^{2}}\frac{\partial^{2}}{\partial \theta^{2}}-\frac{1}{r}\frac{\partial }{\partial r}\right)\left[\sin\theta \frac{d^{2}f_{1}^{+/-}(r)}{dr^{2}}\right]\\ +\frac{1}{E^{+/-}}\left(\frac{\partial^{2}}{\partial r^{2}}+\frac{2}{r}\frac{\partial }{\partial r}\right)\left[\sin\theta \frac{d^{2}f_{1}^{+/-}(r)}{dr^{2}}\right]-\frac{\mu }{E^{+/-}}\left(\frac{\partial^{2}}{\partial r^{2}}+\frac{2}{r}\frac{\partial }{\partial r}\right)\left[\frac{\sin\theta }{r}\frac{df_{1}^{+/-}(r)}{dr}-\frac{\sin\theta }{r^{2}}f_{1}^{+/-}(r)\right]\\ -\frac{2(1+\mu )}{E^{+/-}}\left(\frac{1}{r^{2}}\frac{\partial }{\partial \theta }+\frac{1}{r}\frac{\partial^{2}}{\partial r\partial \theta }\right)\left[\frac{\cos\theta }{r^{2}}f_{1}^{+/-}(r)-\frac{\cos\theta }{r}\frac{df_{1}^{+/-}(r)}{dr}\right]\\ -\frac{\mu }{E^{+/-}}\left(\frac{1}{r^{2}}\frac{\partial^{2}}{\partial \theta^{2}}-\frac{1}{r}\frac{\partial }{\partial r}\right)\left[\frac{d^{2}f_{2}^{+/-}(r)}{dr^{2}}\right]+\alpha \left(\frac{1}{r^{2}}\frac{\partial^{2}}{\partial \theta^{2}}-\frac{1}{r}\frac{\partial }{\partial r}\right)\left[T\left(r\right)\right]\\ +\frac{1}{E^{+/-}}\left(\frac{\partial^{2}}{\partial r^{2}}+\frac{2}{r}\frac{\partial }{\partial r}\right)\left[\frac{d^{2}f_{2}^{+/-}(r)}{dr^{2}}\right]+\frac{1}{E^{+/-}}\left(\frac{1}{r^{2}}\frac{\partial^{2}}{\partial \theta^{2}}-\frac{1}{r}\frac{\partial }{\partial r}\right)\left[\frac{1}{r}\frac{df_{2}^{+/-}(r)}{dr}\right]\\ -\frac{\mu }{E^{+/-}}\left(\frac{\partial^{2}}{\partial r^{2}}+\frac{2}{r}\frac{\partial }{\partial r}\right)\left[\frac{1}{r}\frac{df_{2}^{+/-}(r)}{dr}\right]+\alpha \left(\frac{\partial^{2}}{\partial r^{2}}+\frac{2}{r}\frac{\partial }{\partial r}\right)\left[T\left(r\right)\right]=0 \end{array}.$$

The left of Equation (9) seems to be complicated and hard to deal with, containing the partial differential with respect to *r* and *θ*. But after simple computation, we can definitely get an expression like the form *A*sin*θ + B*, in which *A* and *B* stand for two functions only related to *r*. In this case, for any *θ*, if this expression is equal to zero, that is, *A*sin*θ* + *B* = 0, obviously, we have *A* = 0 and *B* = 0. In fact, this process is referred to as the separation of variables in mathematics. According to this conclusion, for any *θ*, Equation (9) always holds; thus we have

(10) $$\begin{array}{l} -\frac{\mu }{E^{+/-}}\left(-\frac{1}{r}\frac{\partial }{\partial r}\right)\left[\frac{d^{2}f_{2}^{+/-}(r)}{dr^{2}}\right]+\alpha \left(-\frac{1}{r}\frac{\partial }{\partial r}\right)\left[T\left(r\right)\right]\\ +\frac{1}{E^{+/-}}\left(\frac{\partial^{2}}{\partial r^{2}}+\frac{2}{r}\frac{\partial }{\partial r}\right)\left[\frac{d^{2}f_{2}^{+/-}(r)}{dr^{2}}\right]+\frac{1}{E^{+/-}}\left(-\frac{1}{r}\frac{\partial }{\partial r}\right)\left[\frac{1}{r}\frac{df_{2}^{+/-}(r)}{dr}\right]\\ -\frac{\mu }{E^{+/-}}\left(\frac{\partial^{2}}{\partial r^{2}}+\frac{2}{r}\frac{\partial }{\partial r}\right)\left[\frac{1}{r}\frac{df_{2}^{+/-}(r)}{dr}\right]+\alpha \left(\frac{\partial^{2}}{\partial r^{2}}+\frac{2}{r}\frac{\partial }{\partial r}\right)\left[T\left(r\right)\right]=0 \end{array}$$

and

(11) $$\begin{array}{l} \frac{1}{E^{+/-}r^{2}}\left[\frac{1}{r^{2}}f_{1}^{+/-}(r)-\frac{1}{r}\frac{df_{1}^{+/-}(r)}{dr}\right]+\frac{\mu }{E^{+/-}r^{2}}\left[\frac{d^{2}f_{1}^{+/-}(r)}{dr^{2}}\right]\\ +\frac{1}{E^{+/-}}\left(\frac{1}{r}\frac{\partial }{\partial r}\right)\left[\frac{1}{r^{2}}f_{1}^{+/-}(r)-\frac{1}{r}\frac{df_{1}^{+/-}(r)}{dr}\right]\\ +\frac{\mu }{E^{+/-}}\left(\frac{1}{r}\frac{\partial }{\partial r}\right)\left[\frac{d^{2}f_{1}^{+/-}(r)}{dr^{2}}\right]+\frac{1}{E^{+/-}}\left(\frac{\partial^{2}}{\partial r^{2}}+\frac{2}{r}\frac{\partial }{\partial r}\right)\left[\frac{d^{2}f_{1}^{+/-}(r)}{dr^{2}}\right]\\ -\frac{\mu }{E^{+/-}}\left(\frac{\partial^{2}}{\partial r^{2}}+\frac{2}{r}\frac{\partial }{\partial r}\right)\left[\frac{1}{r}\frac{df_{1}^{+/-}(r)}{dr}-\frac{1}{r^{2}}f_{1}^{+/-}(r)\right]\\ +\frac{2(1+\mu )}{E^{+/-}}\left(\frac{1}{r^{2}}+\frac{1}{r}\frac{\partial }{\partial r}\right)\left[\frac{1}{r^{2}}f_{1}^{+/-}(r)-\frac{1}{r}\frac{df_{1}^{+/-}(r)}{dr}\right]=0 \end{array}.$$

For the two ends of Equation (10), multiplying *r*d*r* and integrating with respect to *r*, we have

(12) $$\frac{\left(1+\mu \right)r}{E^{+/-}}\frac{d}{dr}\left[\frac{1}{r}\frac{df_{2}^{+/-}(r)}{dr}\right]+\frac{r}{E^{+/-}}\frac{d}{dr}\left[\frac{d^{2}f_{2}^{+/-}(r)}{dr^{2}}-\frac{\mu }{r}\frac{df_{2}^{+/-}(r)}{dr}+\alpha TE^{+/-}\right]=A^{+/-}.$$

in which *A*^+/−^ is an integral constant. Next, for the two ends of Equation (12), we make the same mathematical operation, that is, multiplying *r*^−1^d*r* and integrating with respect to *r*:

(13) $$\frac{1}{E^{+/-}}\left[\frac{1}{r}\frac{df_{2}^{+/-}(r)}{dr}\right]+\frac{1}{E^{+/-}}\left[\frac{d^{2}f_{2}^{+/-}(r)}{dr^{2}}+\alpha TE^{+/-}\right]=A^{+/-}\ln r+B^{+/-}.$$

in which *B*^+/−^ is another integral constant. After the simplification, we have

(14) $$\frac{1}{r}\frac{d}{dr}\left[r\frac{df_{2}^{+/-}(r)}{dr}\right]=A^{+/-}\ln r+B^{+/-}-\alpha TE^{+/-}.$$

Note that in Equation (14), because *E*^+/−^ is also a constant, the original integral terms *A*^+/−^*E*^+/−^ and *B*^+/−^*E*^+/−^ have been changed to the new *A*^+/−^ and *B*^+/−^ for convenience.

For the two ends of Equation (14), multiplying *r*d*r* and integrating with respect to *r*, and then multiplying *r*^−1^d*r* and integrating with respect to *r*, we have (note that *T* = *T*(*r*))

(15) $$f_{2}^{+/-}(r)=-\alpha E^{+/-}\int \frac{1}{r}\int Trdrdr+A_{2}^{+/-}\ln r+B_{2}^{+/-}r^{2}\ln r+C_{2}^{+/-}r^{2}+D_{2}^{+/-}.$$

in which the integral

(16) $$\int r\ln rdr=\frac{r^{2}}{2}\ln r-\frac{1}{4}r^{2}+C,$$

is used, and the integral constants $A_{2}^{+/-},B_{2}^{+/-},C_{2}^{+/-}$, and $D_{2}^{+/-}$ have been combined and simplified.

Similarly, for Equation (11), we have the same operation and obtain

(17) $$f_{1}^{+/-}(r)=A_{1}^{+/-}r^{3}+B_{1}^{+/-}/r+C_{1}^{+/-}r\ln r+D_{1}^{+/-}r.$$

in which the integral constants $A_{1}^{+/-},B_{1}^{+/-},C_{1}^{+/-}$, and $D_{1}^{+/-}$ have been combined and simplified, and the constant term in $f_{1}^{+/-}(r)$ has been omitted because there is no influence on the stress.

Substituting Equations (15) and (17) into Equation (4), the stress function expressed in terms of undetermined constants will become

(18) $$\begin{array}{ll} \varphi^{+/-}(r,\theta ) & =\sin\theta \left(A_{1}^{+/-}r^{3}+B_{1}^{+/-}/r+C_{1}^{+/-}r\ln r+D_{1}^{+/-}r\right)\\ & -\alpha E^{+/-}\int \frac{1}{r}\int Trdrdr+A_{2}^{+/-}\ln r+B_{2}^{+/-}r^{2}\ln r+C_{2}^{+/-}r^{2}+D_{2}^{+/-} \end{array}.$$

Substituting Equation (18) into Equation (5), the stresses expressed in terms of undetermined constants become

(19) $$\left\{\begin{array}{ll} \sigma_{r}{}^{+/-} & =\sin\theta \left(2A_{1}^{+/-}r-\frac{2B_{1}^{+/-}}{r^{3}}+\frac{C_{1}^{+/-}}{r}\right)\\ & -\frac{E^{+/-}\alpha }{r^{2}}\left(\int_{r_{1}}^{r}Trdr\right)+\frac{A_{2}^{+/-}}{r^{2}}+B_{2}^{+/-}\left(1+2\ln r\right)+2C_{2}^{+/-}\\ \sigma_{\theta }{}^{+/-} & =\sin\theta \left(6A_{1}^{+/-}r+\frac{2B_{1}^{+/-}}{r^{3}}+\frac{C_{1}^{+/-}}{r}\right)\\ & +\frac{E^{+/-}\alpha }{r^{2}}\left(\int_{r_{1}}^{r}Trdr-Tr^{2}\right)-\frac{A_{2}^{+/-}}{r^{2}}+B_{2}^{+/-}\left(3+2\ln r\right)+2C_{2}^{+/-}\\ \tau_{r\theta }{}^{+/-} & =-\cos\theta \left(2A_{1}^{+/-}r-\frac{2B_{1}^{+/-}}{r^{3}}+\frac{C_{1}^{+/-}}{r}\right) \end{array}\right..$$

According to our previous study [31], due to the temperature variation, *T* is always positive; the thermal stress, in this case, will correspond to the compressive state. Thus, *E*^+/−^ should be changed to *E*^−^. In addition, among the above integral operations, the upper bound must be the variable *r* and the lower bound of the integral can be arbitrarily chosen. If we take different lower bounds, there is only a difference in the integral constant. In our study, the inner and outer radius of the curved beam were denoted by *r*_1_ and *r*_2_, respectively; thus, the lower bound of the integral should be *r*_1_ while an integral constant, *D,* was added to the stress. Finally, we have

(20) $$\left\{\begin{array}{ll} \sigma_{r}{}^{+/-} & =\sin\theta \left(2A_{1}^{+/-}r-\frac{2B_{1}^{+/-}}{r^{3}}+\frac{C_{1}^{+/-}}{r}\right)\\ & -\frac{E^{-}\alpha }{r^{2}}\left(\int_{r_{1}}^{r}Trdr+D\right)+\frac{A_{2}^{+/-}}{r^{2}}+B_{2}^{+/-}\left(1+2\ln r\right)+2C_{2}^{+/-}\\ \sigma_{\theta }{}^{+/-} & =\sin\theta \left(6A_{1}^{+/-}r+\frac{2B_{1}^{+/-}}{r^{3}}+\frac{C_{1}^{+/-}}{r}\right)\\ & +\frac{E^{-}\alpha }{r^{2}}\left(\int_{r_{1}}^{r}Trdr+D-Tr^{2}\right)-\frac{A_{2}^{+/-}}{r^{2}}+B_{2}^{+/-}\left(3+2\ln r\right)+2C_{2}^{+/-}\\ \tau_{r\theta }{}^{+/-} & =-\cos\theta \left(2A_{1}^{+/-}r-\frac{2B_{1}^{+/-}}{r^{3}}+\frac{C_{1}^{+/-}}{r}\right) \end{array}\right..$$

There were, in total, thirteen undetermined constants, and they are $A_{1}^{+/-},B_{1}^{+/-},C_{1}^{+/-}$ and $A_{2}^{+/-},B_{2}^{+/-},C_{2}^{+/-}$ as well as *D*. Due to the fact that the number of undetermined constants is much more than in the case without thermal stress or the case without a bimodular effect, the determination process of these unknown constants is also much more complicated than before. However, by using boundary conditions on the inner and outer edges, as well as continuity conditions on the neutral layer, this problem was still solved successfully.

### 3.2. Boundary Conditions and Continuity Conditions

First, let us consider the main boundary condition on the inner edge of the curved beam. Since the inner side of the curved beam is always in compression, the superscript, ‘+/−’, in Equation (20) is naturally taken as the superscript ‘−’, which gives

(21) $$\sigma_{r}^{-}=0,\;\tau_{r\theta }^{-}=0,\;at\;r=r_{1}.$$

Substituting Equation (20) into Equation (21), we have

(22) $$\left\{\begin{array}{l} \sigma_{r}{}^{-}=\sin\theta \left(2A_{1}^{-}r_{1}-\frac{2B_{1}^{-}}{r_{1}{}^{3}}+\frac{C_{1}^{-}}{r_{1}}\right)+\frac{A_{2}^{-}-E^{-}\alpha D}{r_{1}{}^{2}}+B_{2}^{-}\left(1+2\ln r_{1}\right)+2C_{2}^{-}=0\\ \tau_{r\theta }{}^{-}=-\cos\theta \left(2A_{1}^{-}r_{1}-\frac{2B_{1}^{-}}{r_{1}{}^{3}}+\frac{C_{1}^{-}}{r_{1}}\right)=0 \end{array}\right..$$

For any *θ*, Equation (22) always holds. Obviously, we finally have

(23) $$\left\{\begin{array}{l} 2A_{1}^{-}r_{1}-\frac{2B_{1}^{-}}{r_{1}{}^{3}}+\frac{C_{1}^{-}}{r_{1}}=0\\ \frac{A_{2}^{-}}{r_{1}{}^{2}}+B_{2}^{-}\left(1+2\ln r_{1}\right)+2C_{2}^{-}=\frac{E^{-}\alpha }{r_{1}{}^{2}}D \end{array}\right..$$

Similarly, the main boundary condition on the outer edges gives

(24) $$\sigma_{r}^{+}=0,\tau_{r\theta }^{+}=0,\;at\;r=r_{2}.$$

Note that the outer side of the curved beam is always in tension; thus, the superscript is naturally taken as ‘+’. Substituting Equation (20) into Equation (24), we have

(25) $$\left\{\begin{array}{l} \sigma_{r}^{+}=\sin\theta \left(2A_{1}^{+}r_{2}-\frac{2B_{1}^{+}}{r_{2}{}^{3}}+\frac{C_{1}^{+}}{r_{2}}\right)-\frac{E^{-}\alpha }{r_{2}{}^{2}}J_{1}+\frac{A_{2}^{+}-E^{-}\alpha D}{r_{2}{}^{2}}+B_{2}^{+}\left(1+2\ln r_{2}\right)+2C_{2}^{+}=0\\ \tau_{r\theta }^{+}=-\cos\theta \left(2A_{1}^{+}r_{2}-\frac{2B_{1}^{+}}{r_{2}{}^{3}}+\frac{C_{1}^{+}}{r_{2}}\right)=0 \end{array}\right..$$

Obviously, we have

(26) $$\left\{\begin{array}{l} 2A_{1}^{+}r_{2}-\frac{2B_{1}^{+}}{r_{2}{}^{3}}+\frac{C_{1}^{+}}{r_{2}}=0\\ \frac{A_{2}^{+}}{r_{2}{}^{2}}+B_{2}^{+}\left(1+2\ln r_{2}\right)+2C_{2}^{+}=\frac{E^{-}\alpha }{r_{2}{}^{2}}\left(J_{1}+D\right) \end{array}\right.,$$

in which

(27) $$J_{1}=\int_{r_{1}}^{r_{2}}Trdr.$$

In addition, using the de Saint-Venant Principle at the free end of the beam will give the following three conditions:

(28) $$\left\{\begin{array}{l} \int_{\rho }^{r_{2}}\sigma_{\theta }^{+}dr+\int_{r_{1}}^{\rho }\sigma_{\theta }^{-}dr=0\\ \int_{\rho }^{r_{2}}\sigma_{\theta }^{+}rdr+\int_{r_{1}}^{\rho }\sigma_{\theta }^{-}rdr=0\\ \int_{\rho }^{r_{2}}\tau_{r\theta }^{+}dr+\int_{r_{1}}^{\rho }\tau_{r\theta }^{-}dr=\frac{P}{b} \end{array}\right.,\;at\;\theta =0.$$

Substituting Equation (20) into Equation (28), we have, after the integration operation

(29) $${\left[r\sigma_{r}^{+}\right]}_{\rho }^{r_{2}}+{\left[r\sigma_{r}^{-}\right]}_{r_{1}}^{\rho }={\left.r_{2}\sigma_{r}^{+}\right|}_{r=r_{2}}-{\left.\rho \sigma_{r}^{+}\right|}_{r=\rho }+{\left.\rho \sigma_{r}^{-}\right|}_{r=\rho }-{\left.r_{1}\sigma_{r}^{-}\right|}_{r=r_{1}}=0,$$

(30) $$\begin{array}{l} A_{2}^{+}\ln\frac{r_{2}}{\rho }+B_{2}^{+}\left(r_{2}{}^{2}\ln r_{2}-\rho^{2}\ln\rho \right)+C_{2}^{+}\left(r_{2}{}^{2}-\rho^{2}\right)\\ +A_{2}^{-}\ln\frac{\rho }{r_{1}}+B_{2}^{-}\left(\rho^{2}\ln\rho -r_{1}{}^{2}\ln r_{1}\right)+C_{2}^{-}\left(\rho^{2}-r_{1}{}^{2}\right)=E^{-}\alpha J_{2}+E^{-}\alpha D\ln\frac{r_{2}}{r_{1}} \end{array},$$

and

(31) $$A_{1}^{+}\left(r_{2}^{2}-\rho^{2}\right)+B_{1}^{+}\left(\frac{1}{r_{2}^{2}}-\frac{1}{\rho^{2}}\right)+C_{1}^{+}\ln\frac{r_{2}}{\rho }+A_{1}^{-}\left(\rho^{2}-r_{1}^{2}\right)+B_{1}^{-}\left(\frac{1}{\rho^{2}}-\frac{1}{r_{1}^{2}}\right)+C_{1}^{-}\ln\frac{\rho }{r_{1}}=-\frac{P}{b},$$

in which

(32) $$J_{2}=\int_{r_{1}}^{r_{2}}\frac{1}{r}\int_{r_{1}}^{r_{2}}Trdrdr$$

and Equation (29) have been naturally satisfied according to the above boundary conditions and the subsequent continuity conditions.

At the neutral layer, the continuity conditions give:

(33) $$\sigma_{r}^{+}=\sigma_{r}^{-},\sigma_{\theta }^{+}=\sigma_{\theta }^{-}=0,\;at\;r=\rho .$$

Substituting Equation (20) into Equation (33), we have

(34) $$\begin{array}{l} \sin\theta \left(2A_{1}^{+}\rho -\frac{2B_{1}^{+}}{\rho^{3}}+\frac{C_{1}^{+}}{\rho }\right)+\frac{A_{2}^{+}}{\rho^{2}}+B_{2}^{+}\left(1+2\ln\rho \right)+2C_{2}^{+}\\ =\sin\theta \left(2A_{1}^{-}\rho -\frac{2B_{1}^{-}}{\rho^{3}}+\frac{C_{1}^{-}}{\rho }\right)+\frac{A_{2}^{-}}{\rho^{2}}+B_{2}^{-}\left(1+2\ln\rho \right)+2C_{2}^{-} \end{array}$$

and

(35) $$\begin{array}{l} \sin\theta \left(6A_{1}^{+}\rho +\frac{2B_{1}^{+}}{\rho^{3}}+\frac{C_{1}^{+}}{\rho }\right)+\frac{E^{-}\alpha }{\rho^{2}}\left(\int_{r_{1}}^{\rho }Trdr+D-T\rho^{2}\right)-\frac{A_{2}^{+}}{\rho^{2}}+B_{2}^{+}\left(3+2\ln\rho \right)+2C_{2}^{+}\\ =\sin\theta \left(6A_{1}^{-}\rho +\frac{2B_{1}^{-}}{\rho^{3}}+\frac{C_{1}^{-}}{\rho }\right)+\frac{E^{-}\alpha }{\rho^{2}}\left(\int_{r_{1}}^{\rho }Trdr+D-T\rho^{2}\right)-\frac{A_{2}^{-}}{\rho^{2}}+B_{2}^{-}\left(3+2\ln\rho \right)+2C_{2}^{-}\\ =0 \end{array}.$$

For any *θ*, Equation (34) always holds. Obviously, we have the following two relations:

(36) $$\left\{\begin{array}{l} 2A_{1}^{+}\rho -\frac{2B_{1}^{+}}{\rho^{3}}+\frac{C_{1}^{+}}{\rho }=2A_{1}^{-}\rho -\frac{2B_{1}^{-}}{\rho^{3}}+\frac{C_{1}^{-}}{\rho }\\ \frac{A_{2}^{+}}{\rho^{2}}+B_{2}^{+}\left(1+2\ln\rho \right)+2C_{2}^{+}=\frac{A_{2}^{-}}{\rho^{2}}+B_{2}^{-}\left(1+2\ln\rho \right)+2C_{2}^{-} \end{array}\right..$$

Similarly, for any *θ*, Equation (35) always holds; thus, we have the following three relations:

(37) $$\left\{\begin{array}{l} 6A_{1}^{+}\rho +\frac{2B_{1}^{+}}{\rho^{3}}+\frac{C_{1}^{+}}{\rho }=6A_{1}^{-}\rho +\frac{2B_{1}^{-}}{\rho^{3}}+\frac{C_{1}^{-}}{\rho }=0\\ D=T\left(\rho \right)\rho^{2}-\int_{r_{1}}^{\rho }Trdr\\ -\frac{A_{2}^{+}}{\rho^{2}}+B_{2}^{+}\left(3+2\ln\rho \right)+2C_{2}^{+}=-\frac{A_{2}^{-}}{\rho^{2}}+B_{2}^{-}\left(3+2\ln\rho \right)+2C_{2}^{-}=0 \end{array}\right..$$

Up to now, we obtained Equations (23), (26), (30), (31), (36) and (37), in which there are, in total, thirteen relations containing $A_{1}^{+/-},B_{1}^{+/-},C_{1}^{+/-}$ and $A_{2}^{+/-},B_{2}^{+/-},C_{2}^{+/-}$ as well as *D*. By using these relations, it is possible for us to determine the thirteen undetermined constants. The detailed solving process may be referred to in Appendix A.

## 4. Numerical Simulation and Comparison

The software ABAQUS6.14.4 was used to conduct the numerical simulation, in which the subroutine UMAT was also adopted because there was no bimodular material model in this software. First, we needed to compile the subroutine UMAT for the next call and then establish the numerical computation model, in which the related parameters during the numerical simulation are listed in Table 1. The whole computational process is described as follows.

**Figure 5:**
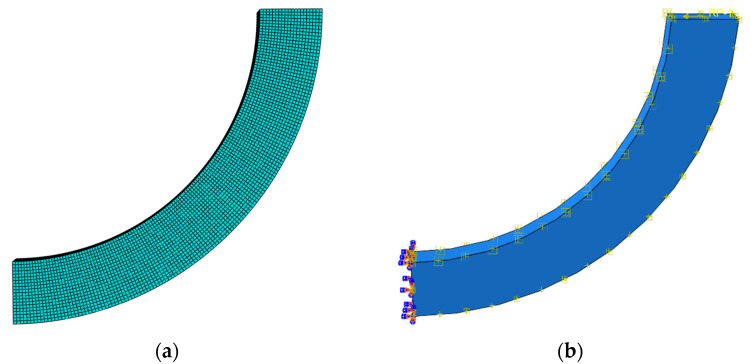
Computational model: (**a**) grid division and (**b**) boundary constraint and loading.

(i) The establishment of a solid model of curved beam, according to the geometrical sizes of the curved beam from Table 1, also see Figure 5a;
(ii) The editing of materials data, including thermal expansion coefficient, the tensile and compressive moduli, and Poisson’s ratios;
(iii) The setting of incremental steps;
(iv) The editing of boundary conditions, one end of the beam is free, and another is fully fixed, see Figure 5b;
(v) The input of the temperature field, in which the temperature rise pattern is defined as *T*(*r*) = *T*_0_ − *T*_0_(*r*−*ρ*)^3^/(0.5 *h*)^3^, *ρ* is the curvature radius of the neutral layer, *h* is the thickness of the curved beam and *T*_0_ is initial temperature rise, as shown in Table 1.
(vi) The input of the end-side concentrated shear load, please see Figure 5b;
(vii) The grid division, in which the mesh was generated using hexahedral elements C3D20 for better accuracy, please see Figure 5a;
(viii) The call of. the UMAT subroutine;
(ix) The output of computational results.

**Figure 6:**
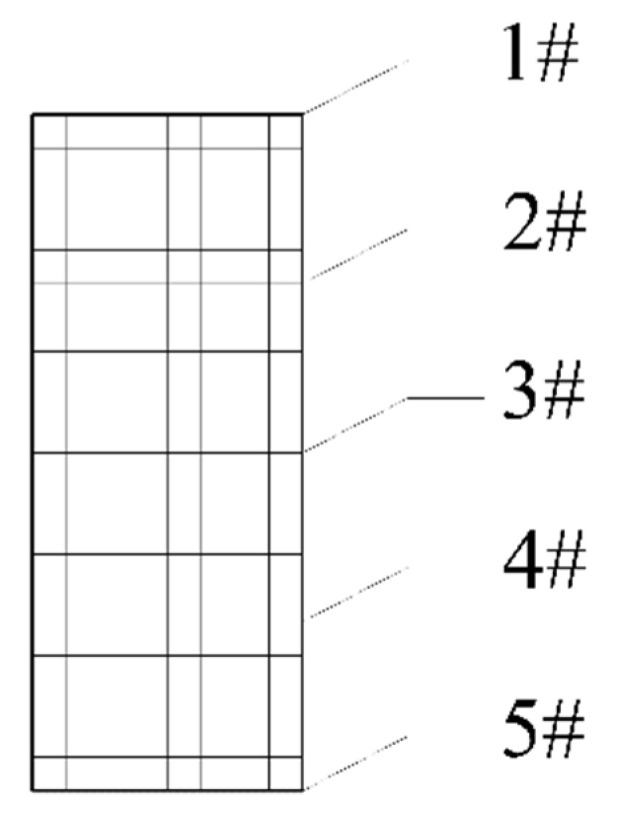
Inspection points on the cross-section of the curved beam.

For the convenience of comparison to the theoretical solution, we selected five inspection points on a certain cross-section of the curved beam. To avoid the negative influences from the fixed end and free end of the beam, the midspan of the curved beam (θ = π/4) was selected. The five inspection points (1# to 5#) were located from the outer edge layer to the inner edge layer, with equal space, as shown in Figure 6.

**Figure 7:**
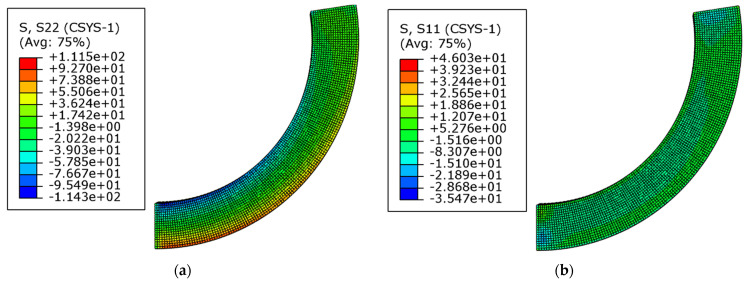
Stress nephogram of the whole curved beam. (**a**) circumferential stress; (**b**) radial stress.

**Figure 8:**
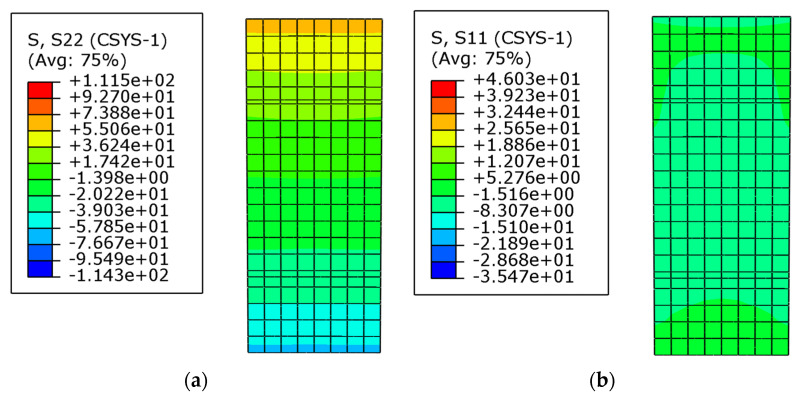
Stress nephogram of the cross-section at *θ* = *π*/4. (**a**) circumferential stress; (**b**) radial stress.

Figure 7 shows the stress nephogram of the whole curved beam, including the circumferential stress and the radial stress. Figure 8 shows the stress nephogram of the cross-section at *θ* = π/4, also including the circumferential stress and the radial stress. From the results of the numerical simulation, the stress values of the five key points were obtained and listed in Table 2 and Table 3, in which Table 2 shows the comparisons of the circumferential stress and Table 3 corresponds to the comparisons of the radial stress. The theoretical results were also computed via Equation (20) and listed in Table 2 and Table 3. It is also easily found from Table 2 and Table 3 that the radial stress is much smaller than the circumferential stress; both the theoretical solution and numerical simulation give the same conclusion, which explains why the radial stress is generally neglected in one-dimensional problems since it is negligibly small.

From Table 2 and Table 3, it is easy to see that the theoretical results are grossly close to the ones from the numerical simulation, with the bigger errors occurring at the inner edge for the circumferential stress. Please refer to inspection point 5# in Table 2; this is mainly due to the influence of the boundary. The overall difference may come from many factors, including the determination of a neutral layer, the differences between the two solution methods themselves, and the precision of the numerical simulation (element selection and grid division, for example). Despite the differences, it is a representation of the real result.

## 5. Bimodular Effect on Stress Distribution

**Figure 9:**
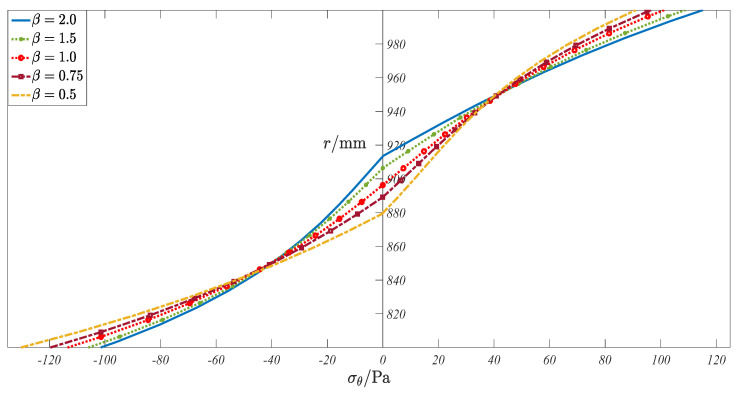
Circumferential stress distribution of five bimodular cases.

In the previous section, the theoretical solution was verified by numerical simulation. In this section, we used the theoretical solution to investigate the bimodular effect on stress distribution. For this purpose, we let the ratio of the tensile modulus to the compressive modulus be parameter *β*, that is,

(38) $$\beta =\frac{E^{+}}{E^{-}},$$

and then we kept the compressive modulus constant (for example, *E*^−^ = 2 × 10^8^ Pa, see Table 1) while changing the tensile modulus (for example, *E*^+^ = 4 × 10^8^ Pa, 3 × 10^8^ Pa, 2 × 10^8^ Pa, 1.5 × 10^8^ Pa and 1 × 10^8^ Pa), that is, *β* is taken as 2.0, 1.5, 1.0, 0.75, and 0.50, according to Equation (38). In the real computation, other given values may refer to Table 1. For different moduli cases, the circumferential stress distribution on the cross-section (which is near the fixed end) is shown in Figure 9, and the stress values of the five inspection points are listed in Table 4 for further reference.

By observing Figure 9, it can be found, first, that all lines are composed of two segments, which reflects well the different moduli characteristics of tension and compression. Secondly, when *β* = 1.0, this case corresponds to the classical material of the same modulus; the expression of the stress component in the tensile region is consistent with that in the compressive region, as shown in Figure 9, in which the curve is continuous and smooth at the neutral layer. For other cases of *β*, the continuity of the curves is still there, but they are not smooth, especially for the cases of *β* = 2 and *β* = 2, in which a clear turn can be observed at the neutral layer. In addition, it can be found that with a decrease in different moduli coefficients, *β*, the tensile elastic modulus gradually decreases. Thus, the height of the tensile zone gradually increases, and the slope of the stress curve gradually decreases (with respect to the horizontal stress axis), while in the compressive zone, it shows completely opposite changes. Such a synchronous change makes the beam maintain the equilibrium of stress on the cross-section.

## 6. Concluding Remarks

In this study, the thermal stress problems of bimodular curved beams under the action of end-side concentrated shear force were analytically and numerically investigated, in which the temperature rise modes of the curved beam in a thermal environment were considered to be arbitrary. The three important conclusions can be drawn as follows.

(i) In the previous problem of pure bending the displacement method based on the displacement potential function was used. While in existing, more general, problems of end-side concentrated shear force, since the displacement method was no longer applicable, the stress method based on compatibility equation was used to solve the problems. The comprehensive application of the two methods improves, to a certain extent, the thermoelastic problem of bimodular materials and structures.
(ii) During the obtainment of the theoretical solution, the number of undetermined constants was much more than in the case without thermal stress or the case without a bimodular effect. But, via stress continuity conditions on the neutral layer and boundary conditions on the inner and outer edges, this problem was still solved successfully.
(iii) The theoretical solution obtained can be reduced to the solution of a bimodular curved beam without thermal stress. At the same time, the numerical simulation for the same problem verifies the correctness of the theoretical solution.

The theoretical solution presented in this study may be used in the refined analysis and optimized design of bimodular curved bars in a thermal environment. The method proposed in this study can be extended to the thermal stress problems of similar structures, namely, arch and shell structures with initial curvature. The relative work is in progress. In addition, the results of this study can be further applied to the analysis of viscoelastic materials, especially under a high-temperature environment [32,33]. Viscoelasticity is the joint property of elasticity and viscosity, and thus it describes materials with both fluid and solid properties simultaneously. If the solid properties of the materials present obviously different elastic properties in tension and compression, their bimodular characteristic should be given some attention.

## Figures and Tables

**Table 1:** Given values in numerical simulation.

Physical Quantities	Taken Values
inner radius, *r*_1_	800 mm
outer radius, *r*_2_	1000 mm
thickness of the curved beam, *h*	200 mm
width of curved beam, *b*	80 mm
tensile modulus, *E*^+^	3 × 10^8^ Pa
compressive modulus, *E*^−^	2 × 10^8^ Pa
tensile Poisson’s ratio, *μ*^+^	0.3
compressive Poisson’s ratio, *μ*^−^	0.3
concentrated shear force*, P*	50 KN
thermal expansion coefficient, *α*	1.6 × 10^−9^/°C
initial temperature rise, *T_0_*	100 °C

**Table 2:** Comparisons of circumferential stress.

Inspection Points	Theoretical Solution (Pa)	Numerical Simulation (Pa)	Absolute Errors (Pa)
1#	69.251	64.677	4.574
2#	22.783	23.779	0.996
3#	−4.049	−4.492	0.443
4#	−25.209	−26.713	1.504
5#	−76.767	−65.952	10.815

**Table 3:** Comparisons of radial stress.

Inspection Points	Theoretical Solution (Pa)	Numerical Simulation (Pa)	Absolute Errors (Pa)
1#	0	−0.171	0.171
2#	−2.359	−2.823	0.464
3#	−3.108	−3.327	0.219
4#	−2.696	−3.528	0.832
5#	0	−0.123	0.123

**Table 4:** Circumferential stress values of inspection points.

Inspection Points	*σ_θ_* (Pa)				
	*β* = 2.0	*β* = 1.5	*β* = 1.0	*β* = 0.75	*β* = 0.5
1#	114.972	108.562	100.952	96.362	90.932
2#	41.095	41.819	41.904	41.543	40.715
3#	−7.198	−3.865	2.765	7.101	11.384
4#	−39.905	−40.226	−40.269	−39.715	−37.683
5#	−101.219	−105.653	−113.143	−119.475	−130.076

## Data Availability

Not applicable.

## Appendix A

In the main text, Equations (23), (26), (30), (31), (36) and (37) were used to determine the thirteen undetermined constants, $A_{1}^{+/-},B_{1}^{+/-},C_{1}^{+/-}$ and $A_{2}^{+/-},B_{2}^{+/-},C_{2}^{+/-}$ as well as *D*. First, among these relations, we found seven equations containing $A_{2}^{+/-},B_{2}^{+/-},C_{2}^{+/-}$ and *D*, and they are from the second relation of Equations (23) and (26), Equation (30), the second relation of Equation (36), as well as the last two relations of Equation (37), which are listed in order as follows:

(A1) $$\left\{\begin{array}{l} \frac{A_{2}^{-}}{r_{1}{}^{2}}+B_{2}^{-}\left(1+2\ln r_{1}\right)+2C_{2}^{-}=\frac{E^{-}\alpha }{r_{1}{}^{2}}D\\ \frac{A_{2}^{+}}{r_{2}{}^{2}}+B_{2}^{+}\left(1+2\ln r_{2}\right)+2C_{2}^{+}=\frac{E^{-}\alpha }{r_{2}{}^{2}}(J_{1}+D)\\ A_{2}^{+}\ln\frac{r_{2}}{\rho }+B_{2}^{+}\left(r_{2}{}^{2}\ln r_{2}-\rho^{2}\ln\rho \right)+C_{2}^{+}\left(r_{2}{}^{2}-\rho^{2}\right)\\ +A_{2}^{-}\ln\frac{\rho }{r_{1}}+B_{2}^{-}\left(\rho^{2}\ln\rho -r_{1}{}^{2}\ln r_{1}\right)+C_{2}^{-}\left(\rho^{2}-r_{1}{}^{2}\right)=E^{-}\alpha J_{2}+E^{-}\alpha D\ln\frac{r_{2}}{r_{1}}\\ \frac{A_{2}^{+}}{\rho^{2}}+B_{2}^{+}\left(1+2\ln\rho \right)+2C_{2}^{+}-\frac{A_{2}^{-}}{\rho^{2}}-B_{2}^{-}\left(1+2\ln\rho \right)-2C_{2}^{-}=0\\ -\frac{A_{2}^{-}}{\rho^{2}}+B_{2}^{-}\left(3+2\ln\rho \right)+2C_{2}^{-}=0\\ -\frac{A_{2}^{+}}{\rho^{2}}+B_{2}^{+}\left(3+2\ln\rho \right)+2C_{2}^{+}=0\\ D=T\left(\rho \right)\rho^{2}-\int_{r_{1}}^{\rho }Trdr \end{array}\right..$$

where

(A2) $$\left\{\begin{array}{l} \frac{E^{-}\alpha }{r_{2}{}^{2}}\left(J_{1}+D\right)=F_{1}\\ \frac{E^{-}\alpha }{r_{1}{}^{2}}D=F_{2}\\ E^{-}\alpha J_{2}+E^{-}\alpha D\ln\frac{r_{2}}{r_{1}}=F_{3} \end{array}\right..$$

$A_{2}^{+/-},B_{2}^{+/-},C_{2}^{+/-}$ may be determined as

(A3) $$\left\{\begin{array}{l} A_{2}^{+}=\frac{-4F_{3}P_{1}}{R}\rho^{2}r_{2}{}^{2}\left(\ln\frac{r_{2}}{\rho }-1\right)+\frac{K_{1}}{R}\\ A_{2}^{-}=\frac{-4F_{3}P_{2}}{R}\rho^{2}r_{1}{}^{2}\left(\ln\frac{r_{1}}{\rho }-1\right)+\frac{K_{2}}{R}\\ B_{2}^{+}=\frac{2F_{3}P_{1}}{R}\left(\rho^{2}+r_{2}{}^{2}\right)+\frac{K_{3}}{R}\\ B_{2}^{-}=\frac{2F_{3}P_{2}}{R}\left(\rho^{2}+r_{1}{}^{2}\right)+\frac{K_{4}}{R}\\ C_{2}^{+}=-\frac{F_{3}P_{1}}{R}\left[\rho^{2}\left(3+2\ln\rho \right)+r_{2}{}^{2}\left(1+2\ln r_{2}\right)\right]+\frac{K_{5}}{R}\\ C_{2}^{-}=-\frac{F_{3}P_{2}}{R}\left[\rho^{2}\left(3+2\ln\rho \right)+r_{1}{}^{2}\left(1+2\ln r_{1}\right)\right]+\frac{K_{6}}{R} \end{array}\right.,$$

in which

(A4) $$\left\{\begin{array}{l} P_{1}=r_{1}{}^{2}-\rho^{2}+2r_{1}{}^{2}\ln\left(\rho /r_{1}\right)\\ P_{2}=r_{2}{}^{2}-\rho^{2}+2r_{2}{}^{2}\ln\left(\rho /r_{2}\right)\\ Q_{1}=4r_{1}{}^{2}\rho^{2}\ln\left(\rho /r_{1}\right)\left[\ln\left(\rho /r_{1}\right)+2\right]+r_{1}{}^{4}+2r_{1}{}^{2}\rho^{2}-3\rho^{4}\\ Q_{2}=4r_{2}{}^{2}\rho^{2}\ln\left(\rho /r_{2}\right)\left[\ln\left(\rho /r_{2}\right)+2\right]+r_{2}{}^{4}+2r_{2}{}^{2}\rho^{2}-3\rho^{4}\\ R=P_{2}Q_{1}-P_{1}Q_{2} \end{array}\right.,$$

and

(A5) $$\begin{array}{l} K_{1}=\rho^{2}r_{2}{}^{2}[4F_{2}r_{1}{}^{4}-F_{1}r_{1}{}^{4}+F_{1}\rho^{2}r_{1}{}^{2}+3F_{1}\rho^{2}r_{2}{}^{2}-4F_{2}\rho^{2}r_{2}{}^{2}\\ -3F_{1}r_{1}{}^{2}r_{2}{}^{2}+8F_{2}r_{1}{}^{4}\ln\rho -4F_{2}r_{1}{}^{4}\ln r_{1}-4F_{2}r_{1}{}^{4}\ln r_{2}+4F_{2}r_{1}{}^{4}\ln^{2}\rho \\ +2F_{1}\rho^{2}r_{1}{}^{2}\ln\rho +2F_{1}\rho^{2}r_{2}{}^{2}\ln\rho -4F_{2}\rho^{2}r_{1}{}^{2}\ln\rho -2F_{1}\rho^{2}r_{1}{}^{2}\ln r_{1}\\ -8F_{1}r_{1}{}^{2}r_{2}{}^{2}\ln\rho -2F_{1}\rho^{2}r_{2}{}^{2}\ln r_{2}+4F_{2}\rho^{2}r_{1}{}^{2}\ln r_{2}+6F_{1}r_{1}{}^{2}r_{2}{}^{2}\ln r_{1}\\ +2F_{1}r_{1}{}^{2}r_{2}{}^{2}\ln r_{2}-4F_{2}r_{1}{}^{4}\ln\rho \ln r_{1}-4F_{2}r_{1}{}^{4}\ln\rho \ln r_{2}+4F_{2}r_{1}{}^{4}\ln r_{1}\ln r_{2}\\ -4F_{1}\rho^{2}r_{1}{}^{2}\ln\left(\rho /r_{1}\right)+4F_{2}\rho^{2}r_{1}{}^{2}\ln\left(\rho /r_{1}\right)-4F_{1}r_{1}{}^{2}r_{2}{}^{2}\ln^{2}\rho \\ +4F_{1}r_{1}{}^{2}r_{2}{}^{2}\ln\rho \ln r_{1}+4F_{1}r_{1}{}^{2}r_{2}{}^{2}\ln\rho \ln r_{2}-4F_{1}r_{1}{}^{2}r_{2}{}^{2}\ln r_{1}\ln r_{2}\\ -4F_{1}\rho^{2}r_{1}{}^{2}\ln\left(\rho /r_{1}\right)\ln\rho +4F_{2}\rho^{2}r_{1}{}^{2}\ln\left(\rho /r_{1}\right)\ln\rho \\ +4F_{1}\rho^{2}r_{1}{}^{2}\ln\left(\rho /r_{1}\right)\ln r_{1}-4F_{2}\rho^{2}r_{1}{}^{2}\ln\left(\rho /r_{1}\right)\ln r_{2}] \end{array}$$

(A6) $$\begin{array}{l} K_{2}=\rho^{2}r_{2}{}^{2}[F_{2}r_{2}{}^{4}-4F_{1}r_{2}{}^{4}+4F_{1}\rho^{2}r_{2}{}^{2}-3F_{2}\rho^{2}r_{1}{}^{2}-F_{2}\rho^{2}r_{2}{}^{2}\\ +3F_{2}r_{1}{}^{2}r_{2}{}^{2}-8F_{1}r_{2}{}^{4}\ln\rho +4F_{1}r_{2}{}^{4}\ln r_{1}+4F_{1}r_{2}{}^{4}\ln r_{2}-4F_{1}r_{2}{}^{4}\ln^{2}\rho \\ +4F_{1}\rho^{2}r_{2}{}^{2}\ln\rho -2F_{2}\rho^{2}r_{1}{}^{2}\ln\rho -2F_{2}\rho^{2}r_{2}{}^{2}\ln\rho -4F_{1}\rho^{2}r_{2}{}^{2}\ln r_{1}\\ +2F_{2}\rho^{2}r_{1}{}^{2}\ln r_{1}+8F_{2}r_{1}{}^{2}r_{2}{}^{2}\ln\rho +2F_{2}\rho^{2}r_{2}{}^{2}\ln r_{2}-2F_{2}r_{1}{}^{2}r_{2}{}^{2}\ln r_{1}\\ -6F_{2}r_{1}{}^{2}r_{2}{}^{2}\ln r_{2}+4F_{1}r_{2}{}^{4}\ln\rho \ln r_{1}+4F_{1}r_{2}{}^{4}\ln\rho \ln r_{2}-4F_{1}r_{2}{}^{4}\ln r_{1}\ln r_{2}\\ +4F_{1}\rho^{2}r_{2}{}^{2}\ln(r_{2}/\rho )-4F_{2}\rho^{2}r_{2}{}^{2}\ln(r_{2}/\rho )+4F_{2}r_{1}{}^{2}r_{2}{}^{2}\ln^{2}\rho \\ -4F_{2}r_{1}{}^{2}r_{2}{}^{2}\ln\rho \ln r_{1}-4F_{2}r_{1}{}^{2}r_{2}{}^{2}\ln\rho \ln r_{2}+4F_{2}r_{1}{}^{2}r_{2}{}^{2}\ln r_{1}\ln r_{2}\\ +4F_{1}\rho^{2}r_{2}{}^{2}\ln(r_{2}/\rho )\ln\rho -4F_{2}\rho^{2}r_{2}{}^{2}\ln(r_{2}/\rho )\ln\rho \\ -4F_{1}\rho^{2}r_{2}{}^{2}\ln(r_{2}/\rho )\ln r_{1}+4F_{2}\rho^{2}r_{2}{}^{2}\ln(r_{2}/\rho )\ln r_{2}] \end{array}$$

(A7) $$\begin{array}{l} K_{3}=[F_{1}\rho^{2}r_{2}{}^{4}+2F_{1}\rho^{4}r_{2}{}^{2}+2F_{2}\rho^{2}r_{1}{}^{4}-2F_{2}\rho^{4}r_{1}{}^{2}-F_{1}r_{1}{}^{2}r_{2}{}^{4}\\ -F_{1}r_{1}{}^{4}r_{2}{}^{2}+2F_{2}r_{1}{}^{4}r_{2}{}^{2}-F_{1}\rho^{2}r_{1}{}^{2}r_{2}{}^{2}-2F_{2}\rho^{2}r_{1}{}^{2}r_{2}{}^{2}+2F_{2}\rho^{2}r_{1}{}^{4}\ln\rho \\ +2F_{2}r_{1}{}^{4}r_{2}{}^{2}\ln\rho +2F_{1}r_{1}{}^{2}r_{2}{}^{4}\ln r_{1}-2F_{2}r_{1}{}^{4}r_{2}{}^{2}\ln r_{1}+2F_{2}\rho^{4}r_{1}{}^{2}\ln(\rho /r_{1})\\ +2F_{1}\rho^{4}r_{2}{}^{2}\ln(r_{2}/\rho )-4F_{1}\rho^{2}r_{1}{}^{2}r_{2}{}^{2}\ln(\rho /r_{1})-2F_{1}\rho^{2}r_{1}{}^{2}r_{2}{}^{2}\ln(r_{2}/\rho )\\ +2F_{2}\rho^{2}r_{1}{}^{2}r_{2}{}^{2}\ln(\rho /r_{1})-2F_{1}\rho^{2}r_{1}{}^{2}r_{2}{}^{2}\ln\rho +2F_{1}\rho^{2}r_{1}{}^{2}r_{2}{}^{2}\ln r_{1}\\ -4F_{1}\rho^{2}r_{1}{}^{2}r_{2}{}^{2}\ln(\rho /r_{1})\ln\rho -4F_{1}\rho^{2}r_{1}{}^{2}r_{2}{}^{2}\ln(r_{2}/\rho )\ln\rho \\ +4F_{1}\rho^{2}r_{1}{}^{2}r_{2}{}^{2}\ln(\rho /r_{1})\ln r_{1}+4F_{1}\rho^{2}r_{1}{}^{2}r_{2}{}^{2}\ln(r_{2}/\rho )\ln r_{1}] \end{array}$$

(A8) $$\begin{array}{l} K_{4}=[-2F_{1}\rho^{2}r_{2}{}^{4}+2F_{1}\rho^{4}r_{2}{}^{2}-F_{2}\rho^{2}r_{1}{}^{4}-2F_{2}\rho^{4}r_{1}{}^{2}-2F_{1}r_{1}{}^{2}r_{2}{}^{4}+F_{2}r_{1}{}^{2}r_{2}{}^{4}\\ +F_{2}r_{1}{}^{4}r_{2}{}^{2}+2F_{1}\rho^{2}r_{1}{}^{2}r_{2}{}^{2}+F_{2}\rho^{2}r_{1}{}^{2}r_{2}{}^{2}-2F_{1}\rho^{2}r_{2}{}^{4}\ln\rho -2F_{1}r_{1}{}^{2}r_{2}{}^{4}\ln\rho \\ +2F_{1}\rho^{2}r_{2}{}^{4}\ln r_{2}+2F_{2}r_{1}{}^{4}r_{2}{}^{2}\ln\rho +2F_{1}r_{1}{}^{2}r_{2}{}^{4}\ln r_{2}-2F_{2}r_{1}{}^{4}r_{2}{}^{2}\ln r_{2}\\ +2F_{2}\rho^{4}r_{1}{}^{2}\ln(\rho /r_{1})+2F_{1}\rho^{4}r_{2}{}^{2}\ln(r_{2}/\rho )+2F_{1}\rho^{2}r_{1}{}^{2}r_{2}{}^{2}\ln(r_{2}/\rho )\\ -2F_{2}\rho^{2}r_{1}{}^{2}r_{2}{}^{2}\ln(\rho /r_{1})-4F_{2}\rho^{2}r_{1}{}^{2}r_{2}{}^{2}\ln(r_{2}/\rho )+2F_{2}\rho^{2}r_{1}{}^{2}r_{2}{}^{2}\ln\rho \\ -2F_{2}\rho^{2}r_{1}{}^{2}r_{2}{}^{2}\ln r_{2}-4F_{2}\rho^{2}r_{1}{}^{2}r_{2}{}^{2}\ln(\rho /r_{1})\ln\rho -4F_{2}\rho^{2}r_{1}{}^{2}r_{2}{}^{2}\ln(r_{2}/\rho )\ln\rho \\ +4F_{2}\rho^{2}r_{1}{}^{2}r_{2}{}^{2}\ln(\rho /r_{1})\ln r_{2}+4F_{2}\rho^{2}r_{1}{}^{2}r_{2}{}^{2}\ln(r_{2}/\rho )\ln r_{2}] \end{array}$$

(A9) $$\begin{array}{l} K_{5}=-[3F_{1}\rho^{4}r_{2}{}^{2}+3F_{2}\rho^{2}r_{1}{}^{4}-3F_{2}\rho^{4}r_{1}{}^{2}-F_{1}r_{1}{}^{4}r_{2}{}^{2}+F_{2}r_{1}{}^{4}r_{2}{}^{2}\\ -2F_{1}\rho^{2}r_{1}{}^{2}r_{2}{}^{2}-F_{2}\rho^{2}r_{1}{}^{2}r_{2}{}^{2}+2F_{1}\rho^{4}r_{2}{}^{2}\ln\rho +5F_{2}\rho^{2}r_{1}{}^{4}\ln\rho -2F_{2}\rho^{4}r_{1}{}^{2}\ln\rho \\ -F_{1}r_{1}{}^{4}r_{2}{}^{2}\ln\rho -3F_{2}\rho^{2}r_{1}{}^{4}\ln r_{1}+F_{1}\rho^{2}r_{2}{}^{4}\ln r_{2}+F_{2}r_{1}{}^{4}r_{2}{}^{2}\ln\rho -F_{1}r_{1}{}^{2}r_{2}{}^{4}\ln r_{2}\\ -F_{2}r_{1}{}^{4}r_{2}{}^{2}\ln r_{1}+2F_{2}r_{1}{}^{4}r_{2}{}^{2}\ln r_{2}+3F_{2}\rho^{4}r_{1}{}^{2}\ln(\rho /r_{1})+3F_{1}\rho^{4}r_{2}{}^{2}\ln(r_{2}/\rho )\\ +2F_{2}\rho^{2}r_{1}{}^{4}\ln^{2}\rho -2F_{2}\rho^{2}r_{1}{}^{4}\ln\rho \ln r_{1}-2F_{1}r_{1}{}^{2}r_{2}{}^{4}\ln\rho \ln r_{2}\\ +2F_{2}r_{1}{}^{4}r_{2}{}^{2}\ln\rho \ln r_{2}+2F_{1}r_{1}{}^{2}r_{2}{}^{4}\ln r_{1}\ln r_{2}-2F_{2}r_{1}{}^{4}r_{2}{}^{2}\ln r_{1}\ln r_{2}\\ -4F_{1}\rho^{2}r_{1}{}^{2}r_{2}{}^{2}\ln(\rho /r_{1})-3F_{1}\rho^{2}r_{1}{}^{2}r_{2}{}^{2}\ln(r_{2}/\rho )+F_{2}\rho^{2}r_{1}{}^{2}r_{2}{}^{2}\ln(\rho /r_{1})\\ +2F_{2}\rho^{4}r_{1}{}^{2}\ln(\rho /r_{1})\ln\rho +2F_{1}\rho^{4}r_{2}{}^{2}\ln(r_{2}/\rho )\ln\rho -2F_{1}\rho^{2}r_{1}{}^{2}r_{2}{}^{2}\ln^{2}\rho \\ -5F_{1}\rho^{2}r_{1}{}^{2}r_{2}{}^{2}\ln\rho +4F_{1}\rho^{2}r_{1}{}^{2}r_{2}{}^{2}\ln r_{1}-2F_{2}\rho^{2}r_{1}{}^{2}r_{2}{}^{2}\ln r_{2}\\ -8F_{1}\rho^{2}r_{1}{}^{2}r_{2}{}^{2}\ln(\rho /r_{1})\ln\rho -8F_{1}\rho^{2}r_{1}{}^{2}r_{2}{}^{2}\ln(r_{2}/\rho )\ln\rho \\ +4F_{1}\rho^{2}r_{1}{}^{2}r_{2}{}^{2}\ln(\rho /r_{1})\ln r_{1}+6F_{1}\rho^{2}r_{1}{}^{2}r_{2}{}^{2}\ln(r_{2}/\rho )\ln r_{1}\\ +2F_{2}\rho^{2}r_{1}{}^{2}r_{2}{}^{2}\ln(\rho /r_{1})\ln r_{2}-4F_{1}\rho^{2}r_{1}{}^{2}r_{2}{}^{2}\ln(\rho /r_{1})\ln^{2}\rho \\ -4F_{1}\rho^{2}r_{1}{}^{2}r_{2}{}^{2}\ln(r_{2}/\rho )\ln^{2}\rho +2F_{1}\rho^{2}r_{1}{}^{2}r_{2}{}^{2}\ln\rho \ln r_{1}\\ +4F_{1}\rho^{2}r_{1}{}^{2}r_{2}{}^{2}\ln(\rho /r_{1})\ln\rho \ln r_{1}+4F_{1}\rho^{2}r_{1}{}^{2}r_{2}{}^{2}\ln(r_{2}/\rho )\ln\rho \ln r_{1}] \end{array}$$

(A10) $$\begin{array}{l} K_{6}=-[-3F_{1}\rho^{2}r_{2}{}^{4}+3F_{1}\rho^{4}r_{2}{}^{2}-3F_{2}\rho^{2}r_{1}{}^{4}-F_{1}r_{1}{}^{2}r_{2}{}^{4}+F_{2}r_{1}{}^{2}r_{2}{}^{4}\\ +F_{1}\rho^{2}r_{1}{}^{2}r_{2}{}^{2}+2F_{2}\rho^{2}r_{1}{}^{2}r_{2}{}^{2}-5F_{1}\rho^{2}r_{2}{}^{4}\ln\rho +2F_{1}\rho^{4}r_{2}{}^{2}\ln\rho -2F_{2}\rho^{4}r_{1}{}^{2}\ln\rho \\ -F_{1}r_{1}{}^{2}r_{2}{}^{4}\ln\rho -F_{2}\rho^{2}r_{1}{}^{4}\ln r_{1}+3F_{1}\rho^{2}r_{2}{}^{4}\ln r_{2}+F_{2}r_{1}{}^{2}r_{2}{}^{4}\ln\rho -2F_{1}r_{1}{}^{2}r_{2}{}^{4}\ln r_{1}\\ +F_{1}r_{1}{}^{2}r_{2}{}^{4}\ln r_{2}+F_{2}r_{1}{}^{4}r_{2}{}^{2}\ln r_{1}+3F_{2}\rho^{4}r_{1}{}^{2}\ln(\rho /r_{1})+3F_{1}\rho^{4}r_{2}{}^{2}\ln(r_{2}/\rho )\\ -2F_{1}\rho^{2}r_{2}{}^{4}\ln^{2}\rho +2F_{1}\rho^{2}r_{2}{}^{4}\ln\rho \ln r_{2}-2F_{1}r_{1}{}^{2}r_{2}{}^{4}\ln\rho \ln r_{1}+2F_{2}r_{1}{}^{4}r_{2}{}^{2}\ln\rho \ln r_{1}\\ +2F_{1}r_{1}{}^{2}r_{2}{}^{4}\ln r_{1}\ln r_{2}-2F_{2}r_{1}{}^{4}r_{2}{}^{2}\ln r_{1}\ln r_{2}+F_{1}\rho^{2}r_{1}{}^{2}r_{2}{}^{2}\ln(r_{2}/\rho )\\ -3F_{2}\rho^{2}r_{1}{}^{2}r_{2}{}^{2}\ln(\rho /r_{1})-4F_{2}\rho^{2}r_{1}{}^{2}r_{2}{}^{2}\ln(r_{2}/\rho )+2F_{2}\rho^{4}r_{1}{}^{2}\ln(\rho /r_{1})\ln\rho \\ +2F_{1}\rho^{4}r_{2}{}^{2}\ln(r_{2}/\rho )\ln\rho +2F_{2}\rho^{2}r_{1}{}^{2}r_{2}{}^{2}\ln^{2}\rho +5F_{2}\rho^{2}r_{1}{}^{2}r_{2}{}^{2}\ln\rho +2F_{1}\rho^{2}r_{1}{}^{2}r_{2}{}^{2}\ln r_{1}\\ -4F_{2}\rho^{2}r_{1}{}^{2}r_{2}{}^{2}\ln r_{2}-8F_{2}\rho^{2}r_{1}{}^{2}r_{2}{}^{2}\ln(\rho /r_{1})\ln\rho -8F_{2}\rho^{2}r_{1}{}^{2}r_{2}{}^{2}\ln(r_{2}/\rho )\ln\rho \\ +2F_{1}\rho^{2}r_{1}{}^{2}r_{2}{}^{2}\ln(r_{2}/\rho )\ln r_{1}+6F_{2}\rho^{2}r_{1}{}^{2}r_{2}{}^{2}\ln(\rho /r_{1})\ln r_{2}+4F_{2}\rho^{2}r_{1}{}^{2}r_{2}{}^{2}\ln(r_{2}/\rho )\ln r_{2}\\ -4F_{2}\rho^{2}r_{1}{}^{2}r_{2}{}^{2}\ln(\rho /r_{1})\ln^{2}\rho -4F_{2}\rho^{2}r_{1}{}^{2}r_{2}{}^{2}\ln(r_{2}/\rho )\ln^{2}\rho -2F_{2}\rho^{2}r_{1}{}^{2}r_{2}{}^{2}\ln\rho \ln r_{2}\\ +4F_{2}\rho^{2}r_{1}{}^{2}r_{2}{}^{2}\ln(\rho /r_{1})\ln\rho \ln r_{2}+4F_{2}\rho^{2}r_{1}{}^{2}r_{2}{}^{2}\ln(r_{2}/\rho )\ln\rho \ln r_{2}] \end{array}.$$

Second, we found six equations containing $A_{1}^{+/-},B_{1}^{+/-},C_{1}^{+/-}$ which are from the first relation of Equations (23) and (26), Equation (31), and the first relation of Equations (36) and (37). They are listed in order as follows:

(A11) $$\left\{\begin{array}{l} 2A_{1}^{-}r_{1}-\frac{2B_{1}^{-}}{r_{1}{}^{3}}+\frac{C_{1}^{-}}{r_{1}}=0\\ 2A_{1}^{+}r_{2}-\frac{2B_{1}^{+}}{r_{2}{}^{3}}+\frac{C_{1}^{+}}{r_{2}}=0\\ A_{1}^{+}\left(r_{2}^{2}-\rho^{2}\right)+B_{1}^{+}\left(\frac{1}{r_{2}^{2}}-\frac{1}{\rho^{2}}\right)+C_{1}^{+}\ln\frac{r_{2}}{\rho }\\ +A_{1}^{-}\left(\rho^{2}-r_{1}^{2}\right)+B_{1}^{-}\left(\frac{1}{\rho^{2}}-\frac{1}{r_{1}^{2}}\right)+C_{1}^{-}\ln\frac{\rho }{r_{1}}=-\frac{P}{b}\\ 2A_{1}^{+}\rho -\frac{2B_{1}^{+}}{\rho^{3}}+\frac{C_{1}^{+}}{\rho }-2A_{1}^{-}\rho +\frac{2B_{1}^{-}}{\rho^{3}}-\frac{C_{1}^{-}}{\rho }=0\\ 6A_{1}^{-}\rho +\frac{2B_{1}^{-}}{\rho^{3}}+\frac{C_{1}^{-}}{\rho }=0\\ 6A_{1}^{+}\rho +\frac{2B_{1}^{+}}{\rho^{3}}+\frac{C_{1}^{+}}{\rho }=0 \end{array}\right.$$

where $A_{1}^{+/-},B_{1}^{+/-},C_{1}^{+/-}$ may be determined as

(A12) $$\left\{\begin{array}{l} A_{1}^{+}=\frac{P\left(\rho^{6}-2\rho^{4}r_{1}{}^{2}+\rho^{4}r_{2}{}^{2}+\rho^{2}r_{1}{}^{4}-2\rho^{2}r_{1}{}^{2}r_{2}{}^{2}+r_{1}{}^{4}r_{2}{}^{2}\right)}{2bS}\\ B_{1}^{+}=-\frac{P\left(3\rho^{6}-6\rho^{4}r_{1}{}^{2}-\rho^{4}r_{2}{}^{2}+3\rho^{2}r_{1}{}^{4}+2\rho^{2}r_{1}{}^{2}r_{2}{}^{2}-r_{1}{}^{4}r_{2}{}^{2}\right)\rho^{2}r_{2}{}^{2}}{2bS}\\ C_{1}^{+}=-\frac{P\left(3\rho^{8}-6\rho^{6}r_{1}{}^{2}+3\rho^{4}r_{1}{}^{4}+\rho^{4}r_{2}{}^{4}-2\rho^{2}r_{1}{}^{2}r_{2}{}^{4}+r_{1}{}^{4}r_{2}{}^{4}\right)}{bS}\\ A_{1}^{-}=\frac{P\left(\rho^{2}+r_{1}{}^{2}\right){\left(\rho^{2}-r_{2}{}^{2}\right)}^{2}}{2bS}\\ B_{1}^{-}=-\frac{P{\left(\rho^{2}-r_{2}{}^{2}\right)}^{2}\left(3\rho^{2}-r_{1}{}^{2}\right)\rho^{2}r_{1}{}^{2}}{2bS}\\ C_{1}^{-}=-\frac{P{\left(\rho^{2}-r_{2}{}^{2}\right)}^{2}\left(3\rho^{4}+r_{1}{}^{4}\right)}{bS} \end{array}\right.,$$

in which

(A13) $$\begin{array}{ll} S & =2\rho^{4}r_{1}{}^{4}-2\rho^{6}r_{1}{}^{2}-2\rho^{4}r_{2}{}^{4}+2\rho^{6}r_{2}{}^{2}+3\rho^{8}\ln\left(\rho /r_{1}\right)+3\rho^{8}\ln\left(r_{2}/\rho \right)\\ & +\rho^{4}r_{1}{}^{4}\ln\left(\rho /r_{1}\right)+3\rho^{4}r_{1}{}^{4}\ln\left(r_{2}/\rho \right)+3\rho^{4}r_{2}{}^{4}\ln\left(\rho /r_{1}\right)-6\rho^{6}r_{1}{}^{2}\ln\left(r_{2}/\rho \right)\\ & -6\rho^{6}r_{2}{}^{2}\ln\left(\rho /r_{1}\right)+\rho^{4}r_{2}{}^{4}\ln\left(r_{2}/\rho \right)+r_{1}{}^{4}r_{2}{}^{4}\ln\left(\rho /r_{1}\right)+r_{1}{}^{4}r_{2}{}^{4}\ln\left(r_{2}/\rho \right)\\ & +2\rho^{2}r_{1}{}^{2}r_{2}{}^{4}-2\rho^{2}r_{1}{}^{4}r_{2}{}^{2}-2\rho^{2}r_{1}{}^{4}r_{2}{}^{2}\ln\left(\rho /r_{1}\right)-2\rho^{2}r_{1}{}^{2}r_{2}{}^{4}\ln\left(r_{2}/\rho \right) \end{array}.$$
